# Supplementary material for: Impacts of mild COVID-19 on elevated use of primary and specialist health care services: A nationwide register study from Norway
Source: PLoS One. 2021 Oct 8;16(10):e0257926. doi: 10.1371/journal.pone.0257926 (PMC8500442; doi:10.1371/journal.pone.0257926)
Supplement: S1 Table — (DOCX) [file pone.0257926.s001.docx]

| **S1 Table. Definitions of the cause-specific diagnosis groups applied** | | | |
| --- | --- | --- | --- |
| **Conditions*** | **ICPC-2 codes used in primary care** | **Short name (in figure)** |  |
| Digestive conditions | D Digestive system | Digestive |  |
| Circulatory conditions | K Circulatory system | Circulatory |  |
| Neurological conditions | N Neurological system | Neurological |  |
| Respiratory conditions | R Respiratory system | Respiratory |  |
| Endocrine, metabolic and nutritional conditions | T Endocrine, metabolic and nutritional system | Endocrine |  |
| Genitourinary conditions | U Urinary system | Urinary |  |
| Eye and ear conditions | F Eye and H Ear | Eye and ear |  |
| Musculoskeletal conditions | L Musculoskeletal system | Musculoskeletal |  |
| Mental conditions | P Psychological, mental and neurodevelopmental | Mental |  |
| Skin conditions | S Skin | Skin |  |
| Blood conditions | B Blood, blood-forming organs, and immune system | Blood |  |
| General and unspecified conditions | A General and unspecified | General and unspecified |  |
| *With conditions we refer to all information that may be included in an ICPC-2 / ICD-10 code: Diseases, disorders, signs, symptoms, and/or complaints as classified by the physician consulted. | | | |
