# Supplementary material for: Impacts of mild COVID-19 on elevated use of primary and specialist health care services: A nationwide register study from Norway
Source: PLoS One. 2021 Oct 8;16(10):e0257926. doi: 10.1371/journal.pone.0257926 (PMC8500442; doi:10.1371/journal.pone.0257926)
Supplement: S2 Table — The DiD estimates captures the change in health care use from 12–1 weeks before PCR test to 1–4, 5–8, and 9–12 weeks after as well as 13–24 weeks after PCR test for patients with mild COVID-19 compared with the change over the same period for patients with no COVID-19. (DOCX) [file pone.0257926.s002.docx]

| S2 Table. Difference-in-differences (DiD) estimates of impacts of COVID-19 on *primary health care use (without obtaining a sick note)* 12-1 weeks before PCR test for SARS-CoV-2 to 1-4, 5-8, and 9-12 weeks after as well as 13-24 weeks after PCR test. The DiD estimates captures the change in health care use from 12-1 weeks before PCR test to 1-4, 5-8, and 9-12 weeks after as well as 13-24 weeks after PCR test for *patients with mild COVID-19* compared with the change over the same period for patients with no COVID-19. | | | | | | | |
| --- | --- | --- | --- | --- | --- | --- | --- |
|  |  | Age 20-44 | | Age 45-69 | | Age 70 and older | |
|  |  | B (95% CI) | % relative diff. (95% CI) | B (95% CI) | % relative diff. (95% CI) | B (95% CI) | % relative diff. (95% CI) |
| Women | |  |  |  |  |  |  |
|  | 1 -4 weeks | 14.430 (13.99,14.88) | 290 (281,299) | 18.360 (17.71,19.01) | 319 (307,330) | 13.66 (12.35,14.98) | 155 (140,170) |
|  | 5-8 weeks | 0.803 ( 0.51, 1.09) | 16 (10,22) | 1.718 ( 1.30, 2.14) | 30 (23,37) | 1.26 ( 0.366, 2.152) | 14 (4,24) |
|  | 9-12 weeks | -0.033 (-0.31, 0.24) | -1 (-6,5) | 0.052 (-0.33, 0.43) | 1 (-6,7) | 0.80 (-0.096, 1.702) | 9 (-1,19) |
|  | 16-24 weeks | -0.851 (-1.07,-0.63) | -17 (-22,-13) | -0.661 (-0.96,-0.36) | -11 (-17,-6) | -0.72 (-1.462, 0.023) | -8 (-17,0) |
| Men | |  |  |  |  |  |  |
|  | 1 -4 weeks | 11.14 (10.79,11.490) | 454 (439,468) | 14.770 (14.22,15.31) | 381 (367,395) | 16.490 (14.90,18.09) | 198 (179,217) |
|  | 5-8 weeks | 0.39 ( 0.19, 0.584) | 16 (8,24) | 0.913 ( 0.59, 1.24) | 24 (15,32) | 1.343 ( 0.25, 2.44) | 16 (3,29) |
|  | 9-12 weeks | -0.22 (-0.41,-0.036) | -9 (-17,-1) | -0.063 (-0.36, 0.23) | -2 (-9,6) | 0.830 (-0.24, 1.90) | 10 (-3,23) |
|  | 16-24 weeks | -0.63 (-0.78,-0.471) | -25 (-32,-19) | -0.643 (-0.89,-0.39) | -17 (-23,-10) | -0.012 (-0.88, 0.85) | 0 (-11,10) |
