# Supplementary material for: Impacts of mild COVID-19 on elevated use of primary and specialist health care services: A nationwide register study from Norway
Source: PLoS One. 2021 Oct 8;16(10):e0257926. doi: 10.1371/journal.pone.0257926 (PMC8500442; doi:10.1371/journal.pone.0257926)
Supplement: S4 Table — The DiD estimates captures the change in health care use from 12–1 weeks before PCR test to 1–4, 5–8, and 9–12 weeks after as well as 13–24 weeks after PCR test for men patients with severe COVID-19 compared with the change over the same period for men patients with no COVID-19. (DOCX) [file pone.0257926.s004.docx]

| S4 Table. Difference-in-differences (DiD) estimates of impacts of COVID-19 on health care use from 12-1 weeks before PCR test for SARS-CoV-2 to 1-4, 5-8, and 9-12 weeks after as well as 13-24 weeks after PCR test. The DiD estimates captures the change in health care use from 12-1 weeks before PCR test to 1-4, 5-8, and 9-12 weeks after as well as 13-24 weeks after PCR test for *men patients with severe COVID-19* compared with the change over the same period for men patients with no COVID-19. | | | | | | | |
| --- | --- | --- | --- | --- | --- | --- | --- |
|  |  | Men 20-44 | | Men 45-69 | | Men 70 and older | |
|  |  | B (95% CI) | % relative diff. (95% CI) | B (95% CI) | % relative diff. (95% CI) | B (95% CI) | % relative diff. (95% CI) |
| Primary care | |  |  |  |  |  |  |
|  | 1 -4 weeks | 20.980 (17.51,24.45) | 686 (573,800) | 25.40 (23.18,27.61) | 555 (507,604) | 13.58 (10.97,16.190) | 167 (135,199) |
|  | 5-8 weeks | 7.267 ( 4.43,10.10) | 238 (145,330) | 9.92 ( 8.16,11.67) | 217 (178,255) | 4.16 ( 2.09, 6.225) | 51 (26,76) |
|  | 9-12 weeks | 1.759 (-0.52, 4.04) | 58 (-17,132) | 5.03 ( 3.49, 6.57) | 110 (76,144) | 0.58 (-1.28, 2.443) | 7 (-16,30) |
|  | 16-24 weeks | 0.098 (-1.57, 1.76) | 3 (-51,58) | 0.20 (-0.86, 1.26) | 4 (-19,27) | -1.35 (-2.74, 0.036) | -17 (-34,0) |
| Specialist care | |  |  |  |  |  |  |
|  | 1 -4 weeks | 15.79 (13.13,18.46) | 1488 (1237,1738) | 18.680 (16.88,20.48) | 1108 (1001,1215) | 14.30 (12.01,16.580) | 339 (285,393) |
|  | 5-8 weeks | 1.33 (-0.51, 3.17) | 126 (-48,299) | 3.381 ( 1.75, 5.02) | 201 (104,297) | 0.19 (-1.91, 2.296) | 5 (-45,54) |
|  | 9-12 weeks | 1.40 (-0.41, 3.21) | 132 (-38,302) | 2.947 ( 1.33, 4.57) | 175 (79,271) | -1.94 (-3.80,-0.083) | -46 (-90,-2) |
|  | 16-24 weeks | -0.25 (-2.25, 1.75) | -24 (-212,165) | -0.085 (-1.41, 1.23) | -5 (-83,73) | -2.80 (-4.51,-1.091) | -66 (-107,-26) |
